# REFLECT—a phase 3 trial comparing efficacy and safety of lenvatinib to sorafenib for the treatment of unresectable hepatocellular carcinoma: an analysis of Japanese subset

**DOI:** 10.1007/s00535-019-01642-1

**Published:** 2019-11-12

**Authors:** Tatsuya Yamashita, Masatoshi Kudo, Kenji Ikeda, Namiki Izumi, Ryosuke Tateishi, Masafumi Ikeda, Hiroshi Aikata, Yasunori Kawaguchi, Yoshiyuki Wada, Kazushi Numata, Yoshitaka Inaba, Ryoko Kuromatsu, Masahiro Kobayashi, Takuji Okusaka, Toshiyuki Tamai, Chifumi Kitamura, Kenichi Saito, Katsuya Haruna, Kiwamu Okita, Hiromitsu Kumada

**Affiliations:** 1 Department of Gastroenterology, Kanazawa University, Kanazawa, Japan; 2 Department of Gastroenterology and Hepatology, Kindai University Faculty of Medicine, Osaka, Japan; 3 Department of Hepatology, Toranomon Hospital, Tokyo, Japan; 4 Department of Gastroenterology and Hepatology, Musashino Red Cross Hospital, Musashino, Japan; 5 Department of Gastroenterology, Graduate School of Medicine, The University of Tokyo, Tokyo, Japan; 6 Department of Hepatobiliary and Pancreatic Oncology, National Cancer Center Hospital East, Kashiwa, Japan; 7 Department of Gastroenterology and Metabolism, Hiroshima University, Hiroshima, Japan; 8 Department of Hepatobiliary and Pancreatology, Saga-Ken Medical Center Koseikan, Saga, Japan; 9 Department of Gastroenterology, Asakura Medical Association Hospital, Asakura, Japan; 10 Department of Hepato-Biliary-Pancreatic Surgery, Clinical Research Institute, National Hospital Organization Kyushu Medical Center, Fukuoka, Japan; 11 Gastroenterological Center, Yokohama City University Medical Center, Yokohama, Japan; 12 Department of Diagnostic and Interventional Radiology, Aichi Cancer Center Hospital, Nagoya, Japan; 13 Division of Gastroenterology, Department of Medicine, Kurume University School of Medicine, Kurume, Japan; 14 Department of Hepatobiliary and Pancreatic Oncology, National Cancer Center Hospital, Tokyo, Japan; 15 Eisai Co., Ltd., Tokyo, Japan; 16 Department of Hepatology, Shunan Memorial Hospital, Kudamatsu, Japan

**Keywords:** REFLECT trial, Hepatocellular carcinoma, Lenvatinib, Sorafenib, Japanese population

## Abstract

**Background:**

A phase 3, multinational, randomized, non-inferiority trial (REFLECT) compared the efficacy and safety of lenvatinib (LEN) and sorafenib (SOR) in patients with unresectable hepatocellular carcinoma (uHCC). LEN had an effect on overall survival (OS) compared to SOR, statistically confirmed by non-inferiority [OS: median = 13.6 months vs. 12.3 months; hazard ratio (HR) 0.92, 95% confidence interval (CI) 0.79–1.06], and demonstrated statistically significant improvements in progression-free survival (PFS) and the objective response rate (ORR) in the overall population. The results of a subset analysis that evaluated the efficacy and safety of LEN and SOR in the Japanese population are reported.

**Methods:**

The intent-to-treat population enrolled in Japan was analyzed.

**Results:**

Of 954 patients in the overall population, 168 Japanese patients were assigned to the LEN arm (*N* = 81) or the SOR arm (*N* = 87). Median OS was 17.6 months for LEN vs. 17.8 months for SOR (HR 0.90; 95% CI 0.62–1.29). LEN showed statistically significant improvements over SOR in PFS (7.2 months vs. 4.6 months) and ORR (29.6% vs. 6.9%). The relative dose intensity of LEN and SOR in the Japanese population was lower than in the overall population. Frequently observed, related adverse events included palmar-plantar erythrodysaesthesia syndrome (PPES), hypertension, decreased appetite, and proteinuria in the LEN arm, and PPES, hypertension, diarrhea, and alopecia in the SOR arm.

**Conclusions:**

The efficacy and safety of LEN in the Japanese population were similar to those in the overall population of REFLECT. With manageable adverse events, LEN is a new treatment option for Japanese patients with uHCC.

**Trial registration ID:**

ClinicalTrials.gov. No. NCT01761266.

**Electronic supplementary material:**

The online version of this article (10.1007/s00535-019-01642-1) contains supplementary material, which is available to authorized users.

## Introduction

Hepatocellular carcinoma (HCC) is the most common primary malignancy of the liver and one of the major causes of cancer-related deaths worldwide [1–4]. A survey in 2015 reported around 28,900 deaths due to hepatocellular carcinoma in Japan [5]. The incidence and mortality rates of HCC are heterogeneous because the prevalence of the risk factors varies among ethnic and geographic populations. The etiology of HCC is primarily related to a chronic infection with hepatitis C virus (HCV) and hepatitis B virus (HBV). Alcohol, aflatoxin, and non-alcoholic steatohepatitis may also cause the disease. In Africa and East Asia, the largest fraction of HCC can be attributed to HBV infection (60%), whereas in North America, Europe, and Japan, chronic hepatitis C appears to be the major risk factor. Treatment options for HCC presently include resection, local ablation, transarterial chemoembolization (TACE), liver transplantation, and systemic therapy. Despite the overall survival rate of HCC patients being considerably improved with advances of diagnostic measures and treatment modalities [3, 6], patients often experience recurrence of the disease and face limited treatment options with advanced disease. This is because HCC frequently develops in patients with chronic liver disease, cirrhosis in particular, which may lead to a limited prognosis after surgical resection [1, 3].

Sorafenib is an inhibitor of multiple protein kinases, including the serine-threonine kinase Raf-1 and tyrosine kinase, vascular endothelial growth factor (VEGF) receptors, and platelet-derived growth factor (PDGF) receptors. This oral medication acts as an antiangiogenic and was shown to be effective for the treatment of patients with unresectable HCC in the Sorafenib Hepatocellular Carcinoma Assessment Randomised Protocol (SHARP) trial [7] and in the phase 3 trial in an Asia-Pacific population [8]. However, because these trials did not include any Japanese patients, the efficacy and safety of the drug as a first-line therapy in the Japanese population were not clear. Sorafenib has been the only approved standard systemic therapy for patients with unresectable HCC. Notably, all phase 3 trials conducted globally with analogous compounds (sunitinib, brivanib, linifanib, and erlotinib plus sorafenib) failed in showing non-inferiority or superiority compared to sorafenib treatment [9–12]. Although regorafenib, nivolumab, pembrolizumab, and cabozantinib are now approved for patients as second-line systemic therapy [13–16] after disease progression with sorafenib treatment, it is important to expand first-line systemic treatment options beyond sorafenib for unresectable HCC.

Lenvatinib is a novel antiangiogenic, orally acting multikinase inhibitor that targets VEGF receptors 1–3, fibroblast growth factor receptors 1–4, PDGF receptor-α, and RET and KIT proto-oncogene products [17, 18]. With the maximum tolerable dose of ~ 25 mg daily suggested for the treatment of solid tumors [19–21], phase 1 and phase 2 trials were conducted including a Japanese population to evaluate exposure to lenvatinib in patients with hepatic impairment. A combination of the trial results and population pharmacokinetic and exposure–response analyses led to recommended lenvatinib doses of 12 mg/day for ≥ 60 kg and 8 mg/day for < 60 kg actual body weight in patients with unresectable HCC with Child–Pugh (C–P) score A [22–27].

Given the observations from these trials, a phase 3, multicenter, randomized, open-label, non-inferiority trial was conducted to compare the efficacy and safety of lenvatinib and sorafenib in first-line therapy for patients with unresectable HCC (REFLECT) [28, 29]^1^ (ClinicalTrials.gov No. NCT01761266). A total of 954 eligible patients were assigned to either the lenvatinib arm or the sorafenib arm at 154 institutional sites in 20 countries and regions throughout the Asia–Pacific including China, Japan, European, and North American regions. Lenvatinib demonstrated a treatment effect on overall survival (OS) compared to sorafenib [median = 13.6 vs. 12.3 months and hazard ratio (HR) = 0.92 with 95% confidence interval (CI) 0.79–1.06] statistically confirmed by non-inferiority; note the predefined upper bound of the 95% CI for non-inferiority was 1.08 [28]. Lenvatinib further demonstrated statistically and clinically significant improvements over sorafenib in progression-free survival (PFS; median = 7.4 vs. 3.7 months), time to progression (TTP; median = 8.9 vs. 3.7 months), and the objective response rate (ORR; 24.1% vs. 9.2%) [28]. Based on the results of the REFLECT study, lenvatinib has expanded the treatment options as a promising first-line therapy for patients with unresectable HCC, and it was recently approved in Japan, the EU, and the USA as a monotherapy for patients with unresectable HCC. The efficacy and safety of lenvatinib and sorafenib in a subset analysis of patients from Japan in the REFLECT study are reported.

## Methods

### Trial design and patient definitions

The REFLECT study was a multicenter, phase 3, randomized, open-label, non-inferiority study. The overall design of the REFLECT study was described in [28], including patient eligibility and procedures for treatment, assessment, and analysis. Briefly, eligible patients had confirmed unresectable HCC based on the American Association for the Study of Liver Diseases criteria [30, 31]. The patients included had one or more measurable target lesions based on mRECIST criteria [26], Barcelona Clinic Liver Cancer (BCLC) stage categorization B (not eligible for or refractory to TACE) or C [29], C-P class A [32], and Eastern Cooperative Oncology Group performance status (ECOG PS) 0 or 1 [33].

Patients provided written, informed consent prior to undergoing any specific procedures, and institutional review boards of the sites individually approved this trial in accordance with the 2008 Declaration of Helsinki and its later amendment, and other relevant laws and regulatory rules.

### Treatments and evaluations

Patients were recruited between March 1, 2013 and July 30, 2015. Eligible patients were randomized in a 1:1 ratio to either the lenvatinib arm or the sorafenib arm with randomization stratification factors of region, extrahepatic spread/macroscopic invasion (yes or no), ECOG PS (0 or 1), and body weight (<60 kg or ≥ 60 kg).

Patients received oral lenvatinib 12 mg/day (for ≥ 60 kg body weight) or 8 mg/day (for < 60 kg body weight) [27, 28] or sorafenib 400 mg twice-daily [7]. During 28-day cycles, dose adjustment was allowed for lenvatinib based on adverse events with reduction to 8 mg or 4 mg per day, 4 mg every other day, and interruption. The sorafenib dose was adjusted according to region-specific prescribing information. At data cut-off on November 13, 2016, the median duration of follow-up was 27.7 months in the lenvatinib arm and 27.2 months in the sorafenib arm.

Tumors were examined by local investigators in accordance with mRECIST for HCC, and mRECIST- and RECIST 1.1-based tumor evaluations [25, 26] were performed by masked central independent imaging review [28]. Tumor assessments were done every 8 weeks (irrespective of dose interruptions) until radiological disease progression.

Pharmacokinetic parameters were derived by a population pharmacokinetic analysis for lenvatinib [28].

### Outcomes and statistical analysis

The primary efficacy endpoint (OS), secondary endpoints (PFS, TTP, ORR, etc.), and the details of the statistical analysis approach for the endpoints were described previously [28].

Safety information was collected throughout the study, and any adverse events (AEs) reported were graded according to the National Cancer Institute Common Terminology Criteria for Adverse Events (CTCAE) version 4.0 [34].

All Japanese subset analyses were performed based on the intent-to-treat population enrolled in Japan. Kaplan–Meier estimates for OS, PFS, and TTP in the two treatment arms are presented, and the differences in PFS and TTP were evaluated using the stratified log–rank test. HRs of lenvatinib vs. sorafenib and their CIs were estimated using a stratified Cox proportional hazards model. The randomization stratification factors were considered as strata. The ORR difference was evaluated using the Cochran–Mantel–Haenszel Chi-square test with randomization stratification factors as strata, and with associated odds ratio (OR) and 95% CI. No multiplicity adjustments were made.

All statistical analyses were performed using SAS version 9.3.

## Results

### Patients’ characteristics

Of the 954 eligible patients randomized to either arm in the REFLECT study [28], 168 Japanese patients (81 receiving lenvatinib and 87 receiving sorafenib) were included in the present analysis. The baseline characteristics were well balanced between the lenvatinib arm and the sorafenib arm within the Japanese population, though etiology of HCV infection and the level of baseline serum α-fetoprotein (AFP) were different (Table 1). Specifically, there were more patients with HCV infection in the sorafenib arm.

**Table 1:** Baseline characteristics of the Japanese patients

Characteristics	Lenvatinib	Sorafenib
	(*N* = 81)	(*N* = 87)
Age (years)		
< 65	18 (22)	30 (34)
65–75	42 (52)	31 (36)
≥ 75	21 (26)	26 (30)
Sex		
Male	65 (80)	72 (83)
Female	16 (20)	15 (17)
Body weight (kg)		
< 60	41 (51)	46 (53)
≥ 60	40 (49)	41 (47)
ECOG PS		
0	76 (94)	75 (86)
1	5 (6)	12 (14)
MPVI		
Yes	15 (19)	15 (17)
No	66 (82)	72 (83)
EHS		
Yes	41 (51)	45 (52)
No	40 (49)	42 (48)
MPVI, EHS, or both		
Yes	49 (60)	52 (60)
No	32 (40)	35 (40)
Child–Pugh class		
A	81 (100)	87 (100)
C-P score 5	58 (72)	67 (77)
C-P score 6	23 (28)	20 (23)
B	0 (0)	0 (0)
BCLC stage		
B	31 (38)	34 (39)
C	50 (62)	53 (61)
Etiology of chronic liver disease		
Hepatitis B	23 (28)	19 (22)
Hepatitis C	37 (46)	49 (56)
Alcohol	10 (12)	6 (7)
Other	5 (6)	2 (2)
Unknown	6 (7)	11 (13)
Baseline total AFP (ng/mL)		
< 200	50 (62)	59 (68)
≥ 200	31 (38)	28 (32)
Median baseline AFP (ng/mL)	57.1	49.8

Numbers are presented as *n* (%) unless otherwise indicated

*ECOG PS* Eastern Cooperative Oncology Group Performance Status, *MPVI* macroscopic portal vein invasion, *EHS* extrahepatic spread, *BCLC stage* Barcelona Clinic Liver Cancer stage, *AFP* alpha-fetoprotein

## Efficacy

**Fig. 1:**
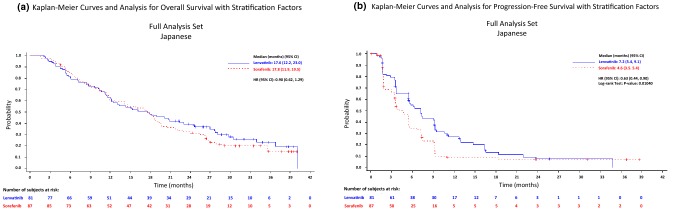
Kaplan–Meier analysis of overall survival (**a**) and progression-free survival (**b**) in the lenvatinib arm and the sorafenib arm of the Japanese unresectable HCC population

**Fig. 2:**
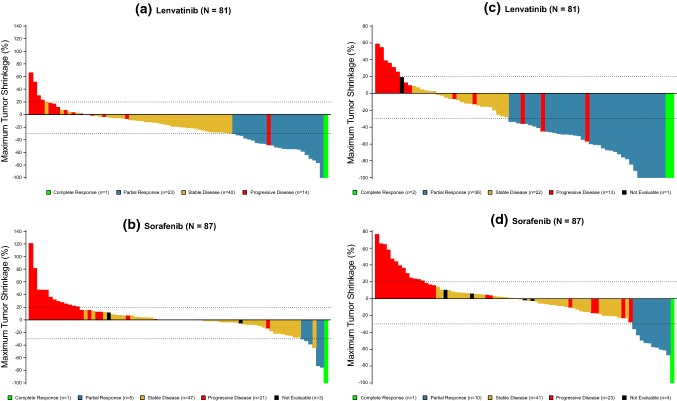
Waterfall plot showing maximum changes in tumor size in the Japanese patients by lenvatinib and sorafenib. Target regions of tumors were examined in the individual patients and assessed for tumor size by local investigators (**a**, **b**) and by masked independent imaging review (**c**, **d**) according to mRECIST. The waterfall plot represents maximum changes in tumor size of each patient receiving lenvatinib (**a**, **c**) and sorafenib (**b**, **d**)

In the Japanese population, the median OS (95% CI) was 17.6 (12.2–23.0) months in the lenvatinib arm and 17.8 (11.9–19.5) months in the sorafenib arm, with an HR (95% CI) of 0.90 (0.62–1.29) (Fig. 1a and Table 2). In the analysis of the secondary efficacy endpoints that were determined by the investigator assessment based on mRECIST, lenvatinib was superior to sorafenib for PFS, with a median of 7.2 vs. 4.6 months and an HR (95% CI) of 0.63 (0.44–0.90; *P* = 0.0104; Fig. 1b and Table 2), and TTP, with a median of 7.2 vs. 4.6 months and an HR of 0.62 (0.43–0.89; *P* = 0.0087). Significant improvement with lenvatinib vs. sorafenib was shown in the ORR (CR + PR), 29.6% vs. 6.9%, with an odds ratio [OR] (95% CI) of 7.03 (2.46–20.09; *P* = 0.0001). One patient in each of the lenvatinib and sorafenib arms had a complete response (CR), and the partial response (PR) rate was several-fold higher in the lenvatinib arm (28.4%) than in the sorafenib arm (5.7%). The disease control rate (DCR; CR + PR + SD) was 79.0% vs. 60.9%, with an OR of 2.42 (1.20–4.87; *P* = 0.0125) (Table 2). Masked independent imaging review according to mRECIST was congruent with the results from the investigators, with longer PFS and TTP and better ORR in the lenvatinib arm than in the sorafenib arm of the Japanese population (median PFS: 7.3 vs. 3.6 months, median TTP: 7.4 vs. 3.6 months, ORR: 46.9% vs. 12.6%). Masked independent imaging review according to RECIST 1.1 also demonstrated the efficacy of lenvatinib over sorafenib. The mRECIST-based masked independent imaging review was further applied to ORR evaluation in a subgroup analysis by baseline characteristics of the Japanese patients (Table S1). The lenvatinib vs. sorafenib profile was generally consistent across the subgroups. A comparison of the antitumor effect of lenvatinib with sorafenib was also schematized by a Waterfall plot of the best response based on mRECIST (Fig. 2).

**Table 2:** Overall survival, progression-free survival, time to progression, objective response rate, and disease control rate in the Japanese population

	Lenvatinib (*N* = 81)	Sorafenib (*N* = 87)	Effect size (95% CI)	*P* value
*Investigator review according to mRECIST*				
OS^a^ (months)	17.6 (12.2–23.0)	17.8 (11.9–19.5)	HR 0.90 (0.62–1.29)	
PFS^a^ (months)	7.2 (5.4–9.1)	4.6 (3.5–5.4)	HR 0.63 (0.44–0.90)	0.0104
TTP^a^ (months)	7.2 (5.4–9.2)	4.6 (3.5–5.4)	HR 0.62 (0.43–0.89)	0.0087
ORR (%, 95% CI)	24 (29.6, 19.7–39.6)	6 (6.9, 1.6–12.2)	OR 7.03 (2.46–20.09)	0.0001
CR	1 (1.2)	1 (1.1)		
PR	23 (28.4)	5 (5.7)		
SD	40 (49.4)	47 (54.0)		
PD	15 (18.5)	23 (26.4)		
UNK/NE	2 (2.5)	11 (12.6)		
DCR (%, 95% CI)	64 (79.0, 70.1–87.9)	53 (60.9, 50.7–71.2)	OR 2.42 (1.20–4.87)	0.0125
*Masked independent imaging review according to mRECIST*				
PFS^a^ (months)	7.3 (5.4–9.4)	3.6 (3.5–5.5)	HR 0.57 (0.38–0.86)	0.0064
TTP^a^ (months)	7.4 (5.4–9.4)	3.6 (3.5–5.5)	HR 0.56 (0.37–0.85)	0.0052
ORR (%, 95% CI)	38 (46.9, 36.0–57.8)	11 (12.6, 5.7–19.6)	OR 5.31 (2.54–11.11)	< 0.0001
CR	2 (2.5)	1 (1.1)		
PR	36 (44.4)	10 (11.5)		
SD	26 (32.1)	41 (47.1)		
PD	13 (16.0)	23 (26.4)		
UNK/NE	13 (16.0)	12 (13.8)		
DCR (%, 95% CI)	64 (79.0, 70.1–87.9)	52 (59.8, 49.5–70.1)	OR 2.62 (1.31–5.24)	0.0056
*Masked independent imaging review according to RECIST 1.1*				
PFS^a^ (months)	7.4 (5.5, 9.4)	3.6 (3.5, 7.2)	HR 0.58 (0.39–0.87)	0.0084
TTP^a^ (months)	7.4 (5.5, 10.6)	3.7 (3.5, 7.2)	HR 0.57 (0.37–0.86)	0.0064
ORR (%, 95% CI)	20 (24.7, 15.3–34.1)	7 (8.0, 2.3–13.8)	OR 3.54 (1.42–8.92)	0.0045
CR	1 (1.2)	0 (0.0)		
PR	19 (23.5)	7 (8.0)		
SD	43 (53.1)	45 (51.7)		
PD	14 (17.3)	23 (26.4)		
UNK/NE	4 (4.9)	12 (13.8)		
DCR (%, 95% CI)	63 (77.8, 68.7–86.8)	52 (59.8, 49.5–70.1)	OR 2.46 (1.23–4.92)	0.0101

Numbers are presented as medians (95% CI) or *n* (%) unless otherwise indicated

*OS* overall survival, *PFS* progression-free survival, *TTP* time to progression, *CR* complete response, *PR* partial response, *SD* stable disease, *PD* progressive disease, *UNK/NE* Unknown or not evaluable, *ORR* objective response rate, *DCR* disease control rate, *OR* odds ratio, *CI* confidence interval, HR hazard ratio

^a^Median OS, PFS, and TTP were calculated by the Kaplan–Meier method

### Safety

All Japanese patients in both the lenvatinib arm and the sorafenib arm experienced AEs and treatment-related AEs (adverse drug reactions; ADRs) (Table S2). AEs and ADRs of grade 3 or higher occurred with similar incidence in the two arms. While the median treatment duration was longer for lenvatinib than for sorafenib (5.7 vs. 3.7 months), adjustment by patient-years [28] gave similar incidence rates of serious AEs and treatment-related serious AEs in both arms (1.1 vs. 0.93 events per patient-years and 0.50 vs. 0.43 events per patient-years, respectively). Table 3 summarizes ADRs reported in the Japanese population with incidence ≥ 20% in either treatment arm. ADRs with grade ≥ 3 were observed in 63.0% of patients receiving lenvatinib and 69.0% of patients receiving sorafenib. Palmar-plantar erythrodysaesthesia syndrome (PPES), hypertension, proteinuria, dysphonia, and diarrhea were the most common in both arms. Decreased appetite and hypothyroidism were more frequent in the lenvatinib arm, and alopecia was more frequent in the sorafenib arm.

**Table 3:** Treatment-related adverse events in the Japanese population

	Lenvatinib (*N* = 81)		Sorafenib (*N* = 87)	
	Any Gr	Gr ≥ 3	Any Gr	Gr ≥ 3
Total treatment-related AEs	81 (100.0)	51 (63.0)	87 (100.0)	60 (69.0)
PPES	42 (51.9)	6 (7.4)	64 (73.6)	15 (17.2)
Hypertension	40 (49.4)	26 (32.1)	42 (48.3)	23 (26.4)
Decreased appetite	39 (48.1)	6 (7.4)	15 (17.2)	0 (0.0)
Proteinuria	37 (45.7)	7 (8.6)	19 (21.8)	1 (1.1)
Dysphonia	35 (43.2)	0 (0.0)	21 (24.1)	0 (0.0)
Hypothyroidism	33 (40.7)	0 (0.0)	4 (4.6)	0 (0.0)
Diarrhea	30 (37.0)	3 (3.7)	32 (36.8)	2 (2.3)
Alopecia	5 (6.2)	0 (0.0)	32 (36.8)	0 (0.0)
Decreased platelet count	23 (28.4)	6 (7.4)	16 (18.4)	9 (10.3)
Edema peripheral	18 (22.2)	1 (1.2)	5 (5.7)	0 (0.0)

Numbers are presented as *n* (%)

The table includes treatment-related adverse events (AEs) of any grade with incidence ≥ 20% observed in either the lenvatinib arm or the sorafenib arm of the Japanese population

*Gr* CTCAE-defined grade, *PPES* palmar-plantar erythrodysaesthesia syndrome

The mean dose intensities of lenvatinib were 6.3 mg/day and 8.5 mg/day in the patients with starting doses of 8 mg and 12 mg, respectively. The mean dose intensity of sorafenib was 558.1 mg/day. Study drugs were reduced, interrupted or discontinued due to ADR occurrence in 61.7% and 59.8%, 56.8% and 46.0%, and 11.1% and 12.6% of lenvatinib-treated patients and of sorafenib-treated patients, respectively. The median time to first dose reduction was 9.9 weeks for lenvatinib and 3.0 weeks for sorafenib.

### Post-study anticancer medications and/or procedures

Following completion/termination of treatment with the trial medications, more than 70% of Japanese patients received post-study anticancer medications and/or procedures in each arm during the survival follow-up period (Table S3). Of the subsequent anticancer medications received by the Japanese patients, sorafenib was used most frequently in both arms (45.7% and 27.6%), followed by antimetabolites (11.1% and 18.4%). Approximately 60% of the Japanese patients underwent post-study anticancer procedures. Commonly performed anticancer procedures were similar in the two arms, including transarterial (chemo) embolization (40% and 44%), followed by hepatic intra-arterial chemotherapy (25% and 24%).

### Pharmacokinetic assessment of lenvatinib

**Fig. 3:**
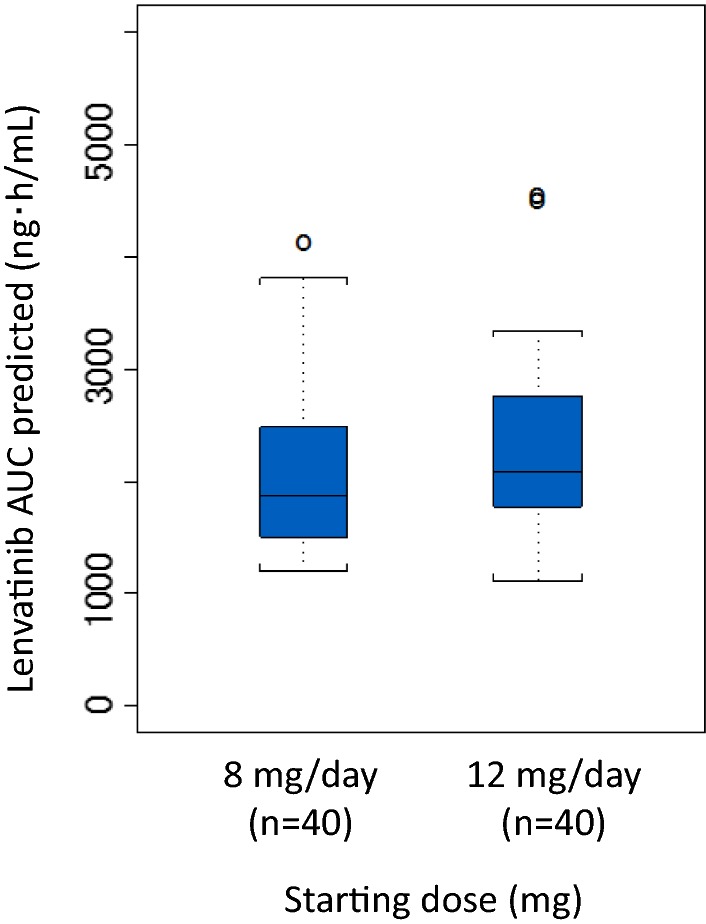
Box plot of lenvatinib AUC predicted in the Japanese population. A population pharmacokinetic analysis was performed in the Japanese population receiving lenvatinib. The box plot represents predicted lenvatinib exposure (AUC) by starting dose (8 mg/day or 12 mg/day). Bar in the box, median value; uppermost and lowermost sides of the box, first and third quartiles, respectively; brackets, range of individual AUCs excluding the outliers denoted by open circles

According to the body weight-based dosing recommendation [27], Japanese patients with a body weight < 60 kg received 8 mg/day lenvatinib as a starting dose, while those with a body weight ≥ 60 kg received 12 mg/day. The median AUC (range) was comparable between the two sub-groups of Japanese patients separated according to body weight, with values of 1868.8 (1197.1–4121.7) ng h/mL and 2077.9 (1116.6–4545.4) ng h/mL, respectively (Fig. 3).

## Discussion

The multinational, phase 3 REFLECT study provided the results of lenvatinib use in patients with unresectable HCC, showing an effect on OS compared to sorafenib that was statistically confirmed by non-inferiority [28, 29]. The results of lenvatinib use in Japanese HCC patients were presented as a subset analysis of the REFLECT study. The Japanese subset was characterized in comparison with the overall population of this trial [28] as aged, and with lower weight, lower ECOG PS score, lower extent of MPVI/EHS, less advanced BCLC stage, and a predominant HCC etiology of chronic infection with HCV rather than HBV. The Japanese subset in this subset analysis was consistent with those reported in the phase 2 trial [24], though there was a higher proportion of patients with ECOG PS score 0 in the present trial. The Japanese patients tended to have lower (<200 ng/mL) levels of serum AFP than the overall population. Within the Japanese subset, chronic HCV infection was observed less frequently in patients receiving lenvatinib than in those receiving sorafenib (46% vs. 56%), as similarly observed, though in a smaller proportion, in the overall population (19% vs. 26%) [28].

The median OS (95% CI) was 17.6 (12.2–23.0) months in the lenvatinib arm vs. 17.8 (11.9–19.5) months in the sorafenib arm of the Japanese subset, giving an HR (95% CI) of lenvatinib vs. sorafenib of 0.90 (0.62–1.29), which was comparable to 0.92 (0.79–1.06) obtained for the overall population [28]. The point estimate of HR (0.90), which was lower than 1, suggests a benefit, although not statistically significant, of lenvatinib use in the treatment of Japanese patients as well. Furthermore, it should be noted that OS was longer in the Japanese subset than in the overall population (17.6 vs. 13.6 months for lenvatinib and 17.8 vs. 12.3 months for sorafenib). This may be explained by more Japanese patients having intermediate stage disease (BCLC-B) at baseline than the overall population (38% vs. 22% in the lenvatinib arm and 39% vs. 19% in the sorafenib arm) and concomitantly fewer having advanced stage disease (BCLC-C; 62% vs. 78% and 61% vs. 81%) and baseline serum AFP < 200 ng/mL (62% vs. 53% and 68% vs. 60%). The difference in the BCLC profile between the Japanese and overall populations was likely to be coincident with the proportion of patients with ECOG PS score 0 (94% vs. 64% and 86% vs. 63%). These considerations are in accordance with a recent study comparing sorafenib and placebo, which demonstrated high AFP and BCLC stage C (vs. B) to be prognostic factors for poor OS [35]. It should be noted that a similar median OS (17.4 months) was obtained with sorafenib in GIDEON [36], in which the Japanese patients (with C-P score A) had baseline characteristics similar to those in the REFLECT study.

In Japan, a nationwide HCC surveillance program has been conducted for decades, encouraging patients to consult their physicians and hence to receive appropriate medications and procedures for HCC treatment at an earlier disease stage. In addition, a high proportion of the Japanese patients compared to the overall population received anticancer medications (49.4% vs. 32.6% in the lenvatinib arm and 49.4% vs. 38.7% in the sorafenib arm) and/or underwent anticancer procedures (55.6% vs. 25.5% and 63.2% vs. 27.3%) during the survival follow-up period after completion or termination of the study treatment. Thus, the long-term surveillance activities and therapeutic improvements achieved in-between have presumably led to the survival as longer OS observed in Japanese patients following lenvatinib (or sorafenib) use than in the overall population [6, 37, 38].

The secondary endpoints including PFS, TTP, and ORR were improved with clinical significance in the Japanese population, when lenvatinib was compared with sorafenib. Similar observations were noted in the overall population [28].

Despite such benefits of lenvatinib use over sorafenib use, there was no significant difference in OS between the two Japanese arms, as observed in the overall population [28]. This may be partly explained by the small sample size of the Japanese subset, but it was more likely due to post-study anticancer therapy provided extensively to this subset. A high proportion of post-sorafenib anticancer therapy was performed in the overall population of the REFLECT trial compared to the previously conducted study [10], and post-progression survival prolonged by such post-study treatments might have led to the minimized difference in the observed overall survival benefit [28]. Thus, the first-line effect of lenvatinib on OS elongation became less visible after the elongated post-progression survival period in the Japanese subset as well.

The median treatment duration was longer in the lenvatinib arm than in the sorafenib arm. Despite this, the total incidence of AEs and ADRs and the incidence of AEs and ADRs of grade 3 or higher were similar in the two treatment arms in the Japanese subset. Hypertension was reported at a higher incidence rate in the Japanese subset than in the overall population in both arms [28]. The mean age was different between the two populations, i.e., elderly patients who have a tendency to have high blood pressure may have contributed to the frequency of hypertension in the Japanese subset. PPES, a representative hand-foot skin reaction known to be associated with sorafenib use, was also the most frequent ADR in the lenvatinib arm, though less than in the sorafenib arm (51.9% vs. 73.6% for any grade; 7.4% vs. 17.2% for Gr ≥ 3). Thus, the patients treated with lenvatinib and sorafenib receiving lenvatinib should be monitored cautiously for these events. Other frequently reported ADRs including proteinuria, decreased appetite, and hypothyroidism in the lenvatinib arm and alopecia and decreased platelet count (Gr ≥ 3) in the sorafenib arm indicate a difference in the safety profile between the two drugs. These ADRs were higher (or similar) in incidence in the Japanese patients compared to the overall population [28]. Thus, it will be important to manage safety in the Japanese patients for ADRs that may arise due to lenvatinib use (and also sorafenib use).

While the mean dose intensity of lenvatinib was lower in the Japanese subset than in the overall population (6.3 vs. 7.0 mg/day and 8.5 vs. 10.5 mg/day in the patients receiving the starting doses of 8 and 12 mg/day, respectively), the proportion of patients who experienced dose reduction and interruption of the drug was higher in the Japanese subset (62% vs. 37% and 57% vs. 40%, respectively), likely due to the higher frequency of certain ADRs. However, the frequency of discontinuations due to ADRs was comparable (11% vs. 9%), suggesting that the ADRs were generally manageable despite their frequent occurrence. In the phase 2 trial, which recruited mostly (>90%) Japanese patients, the starting dose of lenvatinib was set at 12 mg, and 22% of the patients discontinued the trial due to ADRs [24]. The lower rate of ADR-derived discontinuations in the present trial may be explained by the weight-based starting dose of 8 mg/day for < 60 kg and 12 mg/day for ≥ 60 kg. It should be noted that the pharmacokinetic assessment revealed a comparable median AUC between the two subgroups for the starting dose setting. These data strongly suggest that setting the starting dose of lenvatinib based on the patient’s body weight contributed to the proper management of the drug-associated ADRs.

To achieve appropriate ADR management during lenvatinib use, it is important to assess the individual patients prior to the start of treatment for a high risk of developing ADRs and to take appropriate actions, such as dose reduction, dose interruption, and/or supportive therapy to maintain the highest possible dose intensity as high as possible. These measures enable optimization of lenvatinib treatment [38, 39].

In conclusion, a subset analysis of the REFLECT study clearly demonstrated meaningful clinical improvements in PFS and ORR with lenvatinib over sorafenib for the treatment of Japanese patients with HCC. Careful management of AEs is required in clinical practice for long-term use of lenvatinib. Overall, the results presented here provide a basis for lenvatinib use as a first-line therapy, expanding treatment options for Japanese patients with unresectable HCC.

## Electronic supplementary material

Below is the link to the electronic supplementary material.

Supplementary file1 (DOCX 37 kb)

## Footnotes

1 Details of this trial are described in [27], while the abbreviated trial name ‘REFLECT’ is referred to in [28].
