# Supplementary material for: REFLECT—a phase 3 trial comparing efficacy and safety of lenvatinib to sorafenib for the treatment of unresectable hepatocellular carcinoma: an analysis of Japanese subset
Source: J Gastroenterol. 2019 Nov 12;55(1):113–22. doi: 10.1007/s00535-019-01642-1 (PMC6942573; doi:10.1007/s00535-019-01642-1)
Supplement: Supplementary file 1 — Supplementary file1 (DOCX 37 kb) [file 535_2019_1642_MOESM1_ESM.docx]

Table S1. Objective response rate in subgroups of the Japanese population

|  | Events / Patients | | Odds Ratio (95% CI) | ORR (%) | | |  |
| --- | --- | --- | --- | --- | --- | --- | --- |
|  | Lenvatinib (N = 81) | Sorafenib (N = 87) | Lenvatinib vs. Sorafenib | Lenvatinib | | Sorafenib | |
| All | 38/81 | 11/87 | 5.31 (2.54−11.11) | 46.9 | 12.6 | |  |
| Age (years) |  |  |  |  |  | |  |
| <65 | 9/18 | 4/30 | 3.58 (0.81−15.77) | 50.0 | 13.3 | |  |
| ≥65 | 29/63 | 7/57 | 4.77 (1.91−11.92) | 46.0 | 12.3 | |  |
| Sex |  |  |  |  |  | |  |
| Male | 31/65 | 9/72 | 5.62 (2.47−12.76) | 47.7 | 12.5 | |  |
| Female | 7/16 | 2/15 | 3.63 (0.59−22.20) | 43.8 | 13.3 | |  |
| ECOG PS |  |  |  |  |  | |  |
| PS=0 | 35/76 | 9/75 | 5.69 (2.54−12.74) | 46.1 | 12.0 | |  |
| PS=1 | 3/5 | 2/12 | 3.50 (0.57−21.48) | 60.0 | 16.7 | |  |
| Body weight (kg) |  |  |  |  |  | |  |
| <60 | 23/41 | 4/46 | 11.38 (3.35−38.64) | 56.1 | 8.7 | |  |
| ≥60 | 15/40 | 7/41 | 2.79 (1.03−7.53) | 37.5 | 17.1 | |  |
| MPVI, EHS, or both |  |  |  |  |  | |  |
| Yes | 18/49 | 7/52 | 3.39 (1.31−8.76) | 36.7 | 13.5 | |  |
| No | 20/32 | 4/35 | 10.81 (3.21−36.38) | 62.5 | 11.4 | |  |
| AFP at baseline (ng/mL) |  |  |  |  |  | |  |
| <200 | 28/50 | 10/59 | 4.44 (1.89−10.44) | 56 | 16.9 | |  |
| ≥200 | 10/31 | 1/28 | 9.00 (1.33−60.67) | 32.3 | 3.6 | |  |
| Etiology |  |  |  |  |  | |  |
| HBV | 10/25 | 3/22 | 2.89 (0.67−12.53) | 40.0 | 13.6 | |  |
| HCV | 22/38 | 6/51 | 7.11 (2.39−21.20) | 57.9 | 11.8 | |  |
| Alcohol | 4/7 | 1/3 | 2.00 (0.21−19.23) | 57.1 | 33.3 | |  |
| BCLC stage |  |  |  |  |  | |  |
| Stage B | 19/31 | 4/34 | 10.23 (2.99−35.01) | 61.3 | 11.8 | |  |
| Stage C | 19/50 | 7/53 | 3.57 (1.41−9.06) | 38.0 | 13.2 | |  |

Tumor assessment was performed by masked independent imaging review according to mRECIST.

CI, confidence interval; ORR, objective response rate; ECOG PS, Eastern Cooperative Oncology Group Performance Status; MPVI, macroscopic portal vein invasion; EHS, extrahepatic spread; AFP, alpha-fetoprotein; HBV, hepatitis B virus; HCV, hepatitis C virus; BCLC, Barcelona Clinic Liver Cancer.

Table S2. Overview of adverse events

|  | Lenvatinib (N = 81) | Sorafenib (N = 87) |
| --- | --- | --- |
| Any AEs | 81 (100.0) | 87 (100.0) |
| Treatment-related AEs | 81 (100.0) | 87 (100.0) |
| Any grade ≥3 AEs | 63 (77.8) | 66 (75.9) |
| Treatment-related grade ≥3 AEs | 51 (63.0) | 60 (69.0) |
| Any serious AEs | 31 (38.3) | 25 (28.7) |
| Treatment-related serious AEs | 15 (18.5) | 13 (14.9) |

AE, adverse event

Table S3. Post-study anticancer therapy provided to the Japanese population during the survival follow-up period

|  | Lenvatinib (N = 81) | Sorafenib (N = 87) |
| --- | --- | --- |
| Subjects receiving any anticancer  therapy^a^, n (%) | 57 (70.4) | 67 (77.0) |
| Subjects taking any anticancer medication, n (%) | 40 (49.4) | 43 (49.4) |
| Sorafenib | 37 (45.7) | 24 (27.6) |
| Antimetabolites | 9 (11.1) | 16 (18.4) |
| Investigational drugs | 3 (3.7) | 17 (19.5) |
| Subjects that underwent any anticancer procedure, n (%) | 45 (55.6) | 55 (63.2) |
| Transarterial (chemo) embolization | 32 (39.5) | 38 (43.7) |
| Hepatic intra-arterial chemotherapy | 20 (24.7) | 21 (24.1) |
| Radiofrequency ablation | 4 (4.9) | 2 (2.3) |
| Hepatectomy | 1 (1.2) | 1 (1.1) |

^a^ Anticancer therapy denotes either anticancer medication or anticancer procedure.

Numbers (n) denote patients who received anticancer medications or procedures during the survival follow-up period after completion or termination of study treatments.
